# Investigating underlying mechanism in spectral narrowing phenomenon induced by microcavity in organic light emitting diodes

**DOI:** 10.1038/s41467-019-09585-0

**Published:** 2019-04-08

**Authors:** Miaosheng Wang, Jie Lin, Yu-Che Hsiao, Xingyuan Liu, Bin Hu

**Affiliations:** 1 Key Laboratory of Luminescence and Optical Information, Ministry of Education, School of Science, Beijing Jiaotong University, Beijing, 100044 China; 2 Joint Institute for Advanced Materials, Department of Materials Science and Engineering, University of Tennessee, Knoxville, TN 37996 USA; 3 State Key Laboratory of Luminescence and Applications, Changchun Institute of Optics, Fine Mechanics and Physics, Chinese Academy of Sciences, Changchun, 130033 China

## Abstract

This paper reports our experimental studies on the underlying mechanism responsible for electroluminescence spectral narrowing phenomenon in the cavity-based organic light-emitting diodes. It is found that the microcavity generates an emerging phenomenon: a magneto-photoluminescence signal in Poly(9,9-dioctylfluorene-alt-benzothiadiazole) polymer under photoexcitation, which is completely absent when microcavity is not used. This provides an evidence that microcavity leads to the formation of spatially extended states, functioning as the intermediate states prior to the formation of Frenkel excitons in organic materials. This is confirmed by the magneto-electroluminescence solely observed from the cavity-based light-emitting diodes under electrical injection. Furthermore, the narrowed electroluminescence output shows a linear polarization, concurrently occurred with magneto-electroluminescence. This indicates that the spatially extended sates become aligned towards forming coherent light-emitting excitons within the microcavity through optical resonance. Clearly, the spatially extended states present the necessary condition to realize electroluminescence spectral narrowing phenomenon towards lasing actions in cavity-based organic light-emitting diodes.

## Abstract (web summary)

Realizing electrically-pumped lasing actions in microcavity organic light-emitting diodes requires elucidating the origin of spectrally-narrowed electroluminescence amplification. Here, the authors show that the microcavity induces spatially extended states with polaron-pair like characteristics.

## Introduction

Organic semiconductors are known as low-dielectric materials where Frenkel excitons can be formed with high binding energies up to 1 eV^1–3^. Frenkel excitons can normally exhibit high quantum yields through spontaneous emission with large spectral tuning properties by chemically tailoring molecular structures or physically mixing different chromophores in organic semiconductors. Via solution processable and mechanically flexible properties, organic semiconductors have successfully developed unique electroluminescence (EL) actions from thin film organic light-emitting diodes (OLEDs) based on spontaneous emission mechanism^4–6^. However, developing lasing actions from thin film OLEDs has been a long-term challenging endeavor^7–9^. The technical difficulties that prevent the electrically pumped lasing actions are essentially due to the absence of cooperative light-emitting states caused by the energy loss associated with triplets^10, 11^, quenching of light-emitting states from polarons^12^, and insufficient pumping densities from electrical injection^13, 14^. In contrast, optical pumping has led to significant lasing actions in organic semiconductors by using different optical feedback mechanisms based on microcavities and grating designs to realize cooperative Frenkel excitons with significant photoluminescence (PL) spectral narrowing phenomenon as increasing pumping density^9, 15, 16^. However, with the success of optically pumped lasing actions, it is still a challenging issue on whether an optical stimulation is sufficient to generate a cooperative interaction between electrically generated light-emitting states under electrical injection. In general, an electrically pumped lasing action requires an effective mechanism to minimize the optical loss towards developing a cooperative interaction between electrically generated light-emitting states under sufficient electrical injection. It should be noted that using high quality factor (high-Q) microcavity OLEDs has led to a significant EL spectral narrowing phenomenon based on light-emitting molecules of Alq_3_:DCJTI under electrical pumping^17^. The narrowed EL spectrum with the full width at half maximum (FWHM) of 1.95 nm was successfully observed as the injection current was increased to 1000 mA cm^−2^. However, it has been a fundamental question on whether this EL spectral narrowing phenomenon is an indication that the electrically generated excited states become coherent within the microcavity or just a normal microcavity effect. The normal optical microcavity effect originates from the weak coupling interaction between light-emitting molecules and the confined electromagnetic field introduced by the microcavity that can increase the spontaneous emission rate in a resonant radiation mode while suppressing the non-resonant spontaneous emission. On the other hand, the microcavity can fundamentally change the characteristics of the excited states through optical resonance, generating an EL spectral narrowing phenomenon and providing the necessary condition towards developing electrically pumped lasing actions.

Here, we use both magneto-PL and magneto-EL to address whether the microcavity can influence the characteristics of excited states to understand the basic mechanism responsible for spectral narrowing phenomenon occurring in the poly(9,9-dioctylfluorene-alt-benzothiadiazole) (F8BT) OLEDs with various microcavities. We should note that magneto-PL and magneto-EL can be widely observed when the spatially extended excited states such as polaron pairs, electron-hole pairs, and charge-transfer states are formed with antiparallel and parallel spins in organic light-emitting materials^18–20^. In spatially extended excited states, the spin mixing can be easily realized under the influence of internal magnetic interaction such as spin-orbital coupling, hyperfine coupling, and spin scattering because the exchange interaction, which is against the spin mixing, is normally weak. An external magnetic field can disturb the spin mixing and changes the singlet and triplet populations in spatially extended excited states, consequently presenting magneto-PL and magneto-EL when the spatially extended excited states relax to light-emitting states^19, 20^. In contrast, when spatially extended states are absent, Frenkel excitons, functioning as primary excited states with a strong exchange interaction due to short electron-hole separation distance, do not allow spin mixing between singlets and triplets. In this case, an external magnetic field is not able to change the populations on singlet and triplet Frenkel excitons^18^, leading to negligible magneto-PL and magneto-EL when spatially extended excited states are absent. We should further note that Frenkel excitons are the primary light-emitting states formed in organic materials under optical excitation. As a consequence, organic light-emitting materials do not exhibit any detectable magneto-PL under the low field (<1 T) at room temperature with Frenkel excitons as the primary excited states. However, when the spatially extended excited states are formed in light-emitting donor:acceptor systems, a magneto-PL can be easily observed at room temperature and low field (<200 mT)^21–23^. Therefore, magneto-PL provides a convenient experimental tool to explore whether the light-emitting states possess spatially extended characteristics or localized excitonic wavefunctions in the microcavity to understand the EL spectral narrowing phenomenon. In our experimental design, we select the F8BT thin film with the thickness of 65 nm, which does not show any detectable magneto-PL and magneto-EL without the microcavity, to separately fabricate cavity-based and cavity-free OLEDs. In particular, the microcavities were fabricated with high-Q, intermediate-Q, and low-Q structures. The magneto-PL and magneto-EL measurements were then performed on both cavity-free and cavity-based OLEDs under optical and electrical excitations. We found that an optical excitation leads to a clear magneto-PL signal in the cavity-based OLED as contrarily compared to the non-detectable magneto-PL in cavity-free OLED under photoexcitation. This observation indicates that the microcavity induces the formation of spatially extended excited states, in addition to the light-emitting Frenkel excitons, in the F8BT layer under optical excitation. Furthermore, a similar phenomenon was also observed under electrical injection: the microcavity-based OLED shows an appreciable magneto-EL, indicating the formation of spatially extended excited states, while the cavity-free OLED does not exhibit any detectable magneto-EL, lacking the formation of spatially extended excited states. Our studies indicate that the optical microcavity can indeed influence the characteristics of excited states to generate spatially extended states functioning as intermediate states, leading to the spectral narrowing phenomenon under optical and electrical excitations.

## Results

### Material and microcavity device characterization

**Fig. 1:**
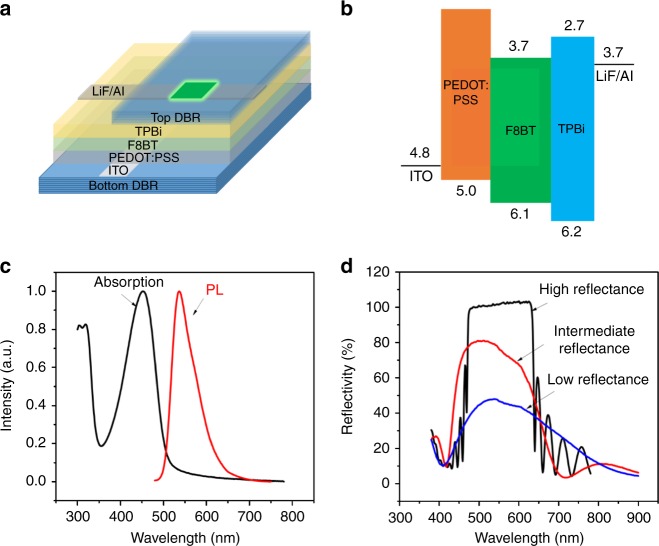
Optical characterizations of cavity-based organic OLEDs. **a** Cavity-based F8BT OLEDs with the structure of Bottom DBR/ITO/PEDOT:PSS/F8BT/TPBi/LiF/Al**/**Top DBR. **b** Energy diagram for F8BT OLEDs. **c** Absorption and PL spectra for F8BT thin film. **d** Reflective spectra at normal incidence angle to bottom DBR used in high-Q, intermediate-Q and low-Q cavity-based OLEDs

**Fig. 2:**
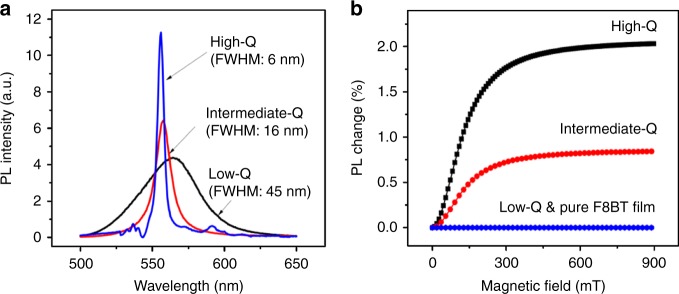
Photoluminescence (PL) and magneto-PL for cavity-based OLEDs. **a** Narrow PL spectra for cavity-based OLEDs with different Q-factors under CW 405 nm laser excitation. **b** Magneto-PL for cavity-based OLEDs with different Q-factors and F8BT thin film at excitation intensity of 1000 mW cm^−2^

Figure 1a, b shows the device structure and energy diagram for the cavity-based OLEDs using the F8BT as the light-emitting medium. Chemical structure of F8BT is shown in Supplementary Fig. 1. The absorption and PL spectra of F8BT thin film are shown in Fig. 1c. Essentially, the microcavity is designed based on the distributed Bragg reflector (DBR) structure: the top mirror consists of Al, ZnS, and MgF_2_ layers, and the bottom mirror combines TiO_2_ and SiO_2_ layers. Within the bottom mirror the TiO_2_/SiO_2_ layers were prepared with 15, 3, and 2 repeating pairs to give different bottom DBR reflectance of 99, 81, and 44 % at 550 nm, leading to high-Q, intermediate-Q, and low-Q microcavities, as shown in Fig. 1d. The Q-factor of the microcavity structures is varied from 94 (high-Q) to 40 (intermediate-Q) and 13 (low-Q). It can be seen in Fig. 2a that using the microcavity can lead to a significantly narrowed PL spectrum under the photoexcitation (1000 mW cm^−2^) of continuous-wave (CW) 405 nm laser beam. The FWHM of PL spectra are determined to be 6, 16, and 45 nm in high-Q, intermediate-Q and low-Q microcavities. Clearly, by increasing the Q factor the PL spectrum becomes gradually narrowed in the microcavity. It is known that an optical excitation generates Frenkel excitons due to low dielectric constants and give rise to broad PL spectra in organic light-emitting materials with inhomogeneous morphologies^2, 3^. Here, it remains as a fundamental question on how the narrowed PL is generated by the Frenkel excitons in the microcavity. Essentially, addressing this question requires an understanding on the effects of microcavity on light-emitting states in the cavity-based F8BT OLEDs.

### Spatially extended states under photoexcitation

To explore the effects of microcavity on light-emitting states, magneto-PL studies were performed by optically exciting the cavity-based F8BT OLEDs with high, intermediate, and low Q-values. We should note that broad PL from Frenkel excitons have a negligible response to an external magnetic field due to strong exchange interaction preserving spin states, lacking magneto-PL under 1 T at room temperature. Without using the microcavity, the F8BT shows this common phenomenon similar to other organic light-emitting materials: non-detectable magneto-PL due to the Frenkel excitons under optical excitation. Figure 2a shows the PL spectra obtained from the microcavity-based OLEDs with high, intermediate, and low Q factors under photoexcitation. The high-Q, intermediate-Q, and low-Q microcavities lead to the FWHM of PL spectra with 6 nm, 16 nm, and 45 nm, respectively. Clearly, the microcavity causes a large PL spectral narrowing phenomenon. This spectral narrowing phenomenon brings about an open question: whether the microcavity influences the characteristics of excited states during the development of spectral narrowing phenomenon? To address this question, we explored magneto-PL from the microcavities with high, intermediate, and low Q factors under optical excitation. When the microcavity is not used, the cavity-free OLED shows a non-detectable, which is a normal phenomenon observed on Frenkel excitons in organic light-emitting materials^18, 20, 24^. Here, we observed a surprising phenomenon by using the microcavity: the narrowed PL shows a magnetic field dependence at low field (<200 mT) and room temperature, leading to a magneto-PL signal (Fig. 2b). Specifically, the PL intensity gradually increases and then becomes saturated with increasing magnetic field, generating a magneto-PL signal at room temperature in cavity-based F8BT OLEDs under photoexcitation. Decreasing the Q factor can directly decrease the magneto-PL signal, simultaneously accompanied with spectral broadening phenomenon. The high-Q and intermediate-Q cavities give the magneto-PL with the amplitudes of 2.0 and 0.8%, respectively. The magneto-PL becomes negligible when further decreasing the Q factor. We should point out that the observed magneto-PL generated in the high-Q microcavity is very similar to that generated by light-emitting charge-transfer states, the spatially extended excited states, formed in donor:acceptor composite^21–23^. Here, the observed magneto-PL clearly indicates that the microcavity can indeed introduce spatially extended excited states, the polaron pairs-like states, in the light-emitting F8BT. Because the spin mixing can easily occur in spatially extended excited states due to weak exchange interaction, an external magnetic field can change the populations on the singlet and triplet spatially extended states through spin mixing, leading to a magneto-PL when the spatially extended states relax to light-emitting Frenkel excitons.

We further discuss whether the observed magneto-PL represents spatially extended excited states by removing the contribution from triplet-triplet annihilation (TTA). It should be mentioned that TTA can occur within Frenkel excitons when two triplet excitons mutually interact within close proximity. The TTA is a bimolecular process which requires a high density of triplet excitons. In general, the TTA is a very limited component in steady-state PL in both organic fluorescent and phosphorescent materials under photoexcitation. As a consequence, both fluorescent and phosphorescent materials do not demonstrate any detectable magneto-PL in steady state at room temperature^18^., In time-dependent measurement where the TTA is selectively monitored, it was found that the TTA gives a negative sign on the magneto-PL/EL signals based on the assumption that a magnetic field decreases the TTA rate constant by perturbing spin interaction^25–28^. Here, our magneto-PL with positive sign observed in cavity-based F8BT OLEDs does not suggest the TTA occurring in F8BT within the microcavity. Furthermore, the literature publications have shown controversy information on whether the TTA is occurred in the F8BT based OLEDs with favor^29, 30^ and in favor arguments^31^. Here, our PL lifetime studies provide additional evidence to indicate that the TTA is not occurred: the F8BT within the microcavity shows a short PL lifetime (0.88 ns) (Supplementary Fig. 2). Nevertheless, it is very clear that without microcavity, no magneto-PL can be detected in the F8BT, similar phenomenon commonly observed in organic materials under photoexcitation, indicating that the Frenkel excitons are primary light-emitting states in the pristine F8BT. However, with microcavity, our magneto-PL indicates that spatially extended excited states are indeed formed with the characteristics similar to polaron pairs, functioning as intermediate states within cavity-based F8BT OLEDs. This creates an interesting question on how the spatially extended states are induced by the microcavity. Here, we consider the following possibility to understand the effects of microcavity on light-emitting states based on magneto-PL with spectral narrowing phenomenon. When the optical confinement is introduced to form an optical field in the microcavity, the Frenkel excitons placed in an optical field develop into resonant states, leading to spatially extended states with the characteristics similar to polaron pairs. This means that the optical field can extend the wavefunctions of Frenkel excitons in cooperative manner, forming resonant spatially extended states in the microcavity. The resonant spatially extended states with cooperative manor can essentially form coherent light-emitting Frenkel excitons through recombination, generating a narrowed PL. Although this possibility demands further investigation, it is clear that the microcavity introduces the additional excited states with the characteristics similar to polaron pairs, accompanied with spectral narrowing phenomenon, in the cavity-based F8BT OLEDs under photoexcitation, according to the observed magneto-PL.

### Spatially extended states under electrical excitation

**Fig. 3:**
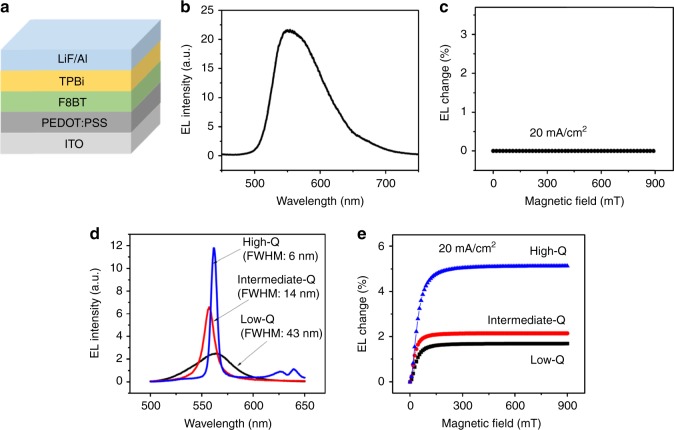
Electroluminescence (EL) in cavity-free and cavity-based OLEDs. **a** The device structure of cavity-free F8BT OLED. **b** Broad EL spectrum for cavity-free OLED. **c** Negligible magneto-EL at different injection currents for broad EL in cavity-free OLED. **d** Narrow EL spectra for cavity-based OLEDs with different Q-factors. **e** Magneto-EL for cavity-based OLEDs with different Q-factors at constant injection current (20 mA cm^−2^)

Now we confirm the characteristics of spatially extended states within the cavity-based F8BT OLED under electrical injection by using magneto-EL measurement at constant-current mode. The maximum external quantum efficiencies (EQE) for cavity-free and high Q cavity-based F8BT OLEDs are 4.45 and 0.92% (Supplementary Fig. 3), respectively. It is a common phenomenon that microcavity suppresses the optical output and consequently confines the optical field to realize an optical resonance. Light-emitting intensity and spectral width as function of pumping intensity in high-Q cavity-based F8BT device is shown in Supplementary Fig. 4. We know that polaron pairs can be largely formed towards light-emitting excitons in OLEDs under electrical injection^32, 33^. It has been experimentally observed that an external magnetic field can conveniently change the populations on the singlet and triplet polaron pairs, leading to magneto-EL^18, 34, 35^ and magneto-current^36, 37^ due to largely different recombination rates associated with singlets and triplets. Therefore, magneto-EL can be measured at two different conditions: constant-current and constant-voltage modes, while monitoring the EL intensity with scanning magnetic field^18, 19, 38^. At the constant-current mode, the EL intensity is changed purely by the populations on polaron-pair states through spin mixing caused by magnetic field, leading to spin mixing-based magneto-EL, namely magneto-EL_(mixing)_ (see Supplementary Note 1 for additional discussion on the mechanism of magneto-luminescence). At constant-voltage mode, the EL intensity is changed by both magneto-current and the populations through spin mixing under the influence of magnetic field, generating the combination of spin mixing-based magneto-EL_(mixing)_ and current-based magneto-EL_(current)_. Here, we use magneto-EL at constant-current mode to confirm that the spatially extended states are formed in the microcavity under electrical injection based on whether the spin mixing is occurred. We should point out that magneto-EL can be normally observed in cavity-free OLEDs with the thicker F8BT layer such as 200 nm (Supplementary Fig. 5). However, by carefully selecting the thickness (65 nm) of active F8BT layer, the cavity-free OLEDs with the same device structure, as shown in Fig. 3a, do not show any detectable magneto-EL signal with a broad spectral width (FWHM = 87 nm), as indicated in Fig. 3b, c. The absence of magneto-EL implies that, at this layer thickness, the injected electrons and holes do not have an opportunity to form the spatially extended states and directly recombine into Frenkel excitons. As expected, the electrically generated Frenkel excitons do not demonstrate a magneto-EL signal due to the negligible spin mixing caused by strong exchange interaction. Interestingly, when the microcavity is used with this selected thickness of active layer, a magneto-EL signal can be clearly observed with a narrow EL spectrum (FWHM = 6 nm), as shown in Fig. 3d, e. Furthermore, we can see that increasing the Q factor in the microcavity can appreciably increase the magneto-EL amplitude from 1.7 to 2.1 and 5.1% with the FWHM changed from 43 nm to 14 nm and 6 nm. This magneto-EL confirms that the microcavity can indeed introduce spatially extended states with the characteristics similar to polaron pairs with spectrally narrowed EL under electrical injection.

### Polarization characterization on narrowed EL

**Fig. 4:**
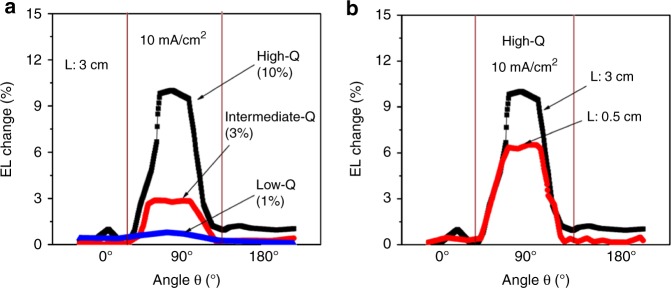
Linear polarization from spectrally narrowed EL in cavity-based OLEDs. θ is the angle between the polarization axis of linear polarizer and ITO stripe in cavity-based OLEDs. **a** Linearly polarized EL measured at 3.0 cm distance from cavity-based OLEDs with different Q-factors. **b** Linearly polarized EL measured at 0.5 cm and 3.0 cm distances for high-Q device

To further investigate the characteristics of spatially extended states induced by the optical field in microcavity, we characterized the polarization of spectrally narrowed EL in the cavity-based OLEDs. We can see in Fig. 4a that the spectrally narrowed EL is essentially a linearly polarized output in the direction parallel to the Al electrode stripe. The linear polarization ratio increases from 1 to 3 and 10% with increasing the Q factor from low-Q, intermediate-Q, and high-Q cavity-based F8BT LEDs at the injection current of 10 mA cm^−2^. Furthermore, the linear polarization of spectrally narrowed EL is a function of detection distance, leading to 6 and 10 at 0.5 cm and 3.0 cm distances vertically from the device surface, as shown in Fig. 4b. It should be noted that EL output is a directional signal from microcavity with the emission concentrated on the cavity axis. At a short distance, the directional beam contains the non-directional light emission from the edge of the working area. As the distance is increased, the non-directional component becomes weaker as compared to the directional signal, which in turn increases the polarization degree of the EL output. Clearly, this linear polarization of spectrally narrowed EL indicates that the light-emitting excitons become spatially aligned in the microcavity under the influence of optical field. Here, we propose that the spatial alignment of light-emitting excitons occurs through the spatially extended states under the influence of microcavity to generate a spectral narrowing phenomenon. Specially, under the influence of optical field in the microcavity, the spatially extended states become aligned with resonant cooperative interaction. When the aligned polaron pairs-like states with cooperative interaction relax into coherent light-emitting excitons, spectrally narrowed EL with linear polarization is developed in the microcavity under electrical injection (Supplementary Fig. 6 and Supplementary Note 2). Therefore, the alignment of spatially extended cooperative states provides the necessary condition to generate coherent light-emitting states for developing potential electrically pumped lasing actions in organic materials.

## Discussion

It is noted that it is difficult to directly detect the excited states within our high-Q microcavity-based OLEDs where EL spectral narrowing phenomenon is occurred. However, we have provided a solid evidence that the spatially extended states are really generated in the microcavity towards developing coherent light-emitting states based on PL and EL observations. We found that the microcavity can generate a magneto-PL signal, accompanied with spectral narrowing phenomenon, in light-emitting F8BT polymer under photoexcitation. This magneto-PL is the unique phenomenon observed from the microcavity F8BT OLED under photoexcitation. This indicates that the microcavity can extend the wavefunctions of optically generated Frenkel excitons through the internal optical field, forming spatially extended excited states with the characteristics similar to polaron pairs. The microcavity-induced spatially extended excited states are confirmed by the experimental observation that lowering the Q factor largely decreases the magneto-PL signal. When the Q-factor of the microcavity structures is varied from 94 (high-Q) to 40 (intermediate-Q) and 13 (low-Q) with the FWHM changing from 6 nm to 16 nm and 45 nm, magneto-PL is decreased from 2.0 to 0.8% and negligible value. Clearly, spatially extended excited states with the characteristics similar to polaron pairs are concurrently occurred with spectral narrowing phenomenon within the microcavity-based F8BT OLED. Furthermore, we observed that introducing the microcavity can enable the magneto-EL under narrowed spectrum after the magneto-EL is disabled under broad spectrum by selecting the active layer thickness of 65 nm in cavity-free OLEDs. This observation indicates that the microcavity can influence electrically generated Frenkel excitons through the optical field, leading to spatially extended excited states in the light-emitting F8BT polymer under electrical pumping. Moreover, the spectrally narrowed EL is found to be a linearly polarized output, indicating that spatially light-emitting states are aligned within cavity-based F8BT OLED. This leads to the hypothesis that the spatially extended excited states induced by the microcavity undergo a cooperative interaction towards developing spectral narrowing phenomenon, presenting the promising condition to realize electrically pumped lasing actions in organic materials.

## Methods

### Device fabrication

The bottom DBR of low-Q microcavity device consists of 2 pairs of periodic TiO_2_ (61.3 nm-thick)/SiO_2_ (99.4 nm-thick) layers underneath the ITO electrode (80 nm-thick). In the intermediate-Q microcavity device, the bottom DBR consists of 3 pairs of periodic TiO_2_ (61.3 nm-thick)/SiO_2_ (99.4 nm-thick) layers underneath the ITO electrode (80 nm-thick). In the high-Q microcavity device the bottom DBR of consists of 15 pairs of periodic TiO_2_ (61.3 nm-thick)/SiO_2_ (99.4 nm-thick) underneath the ITO electrode (80 nm-thick). The TiO_2_, SiO_2_, and ITO layers were deposited by electron beam evaporation at a substrate temperature of 250 °C in an oxygen pressure of 2 × 10^−2^ Pa with deposition rates of 3.5–4.0, 3.0–3.5, and 2.0–2.5 Å s^−1^, respectively. The patterned ITO electrodes on DBR with sheet resistance of ~80 Ω sq^−1^ were treated with the UV-ozone method for 15 min. The PEDOT:PSS (Baytron PVPAl 4083, filtered through a 0.22 μm filter) were spin-coated onto ITO electrodes at 3100 rpm for 30 s. Then the PEDOT:PSS-coated substrates were transferred into a nitrogen-filled glove box (O_2_ < 1 p.p.m., H_2_O < 1 p.p.m.). F8BT (10 mg ml^−1^, o-xylene), was deposited by spin-coating on the PEDOT:PSS layer. The F8BT layer was baked at 120 °C for 10 min. Then, TPBi and LiF layers were deposited using a thermal evaporation system, at deposition rates of 1–2, and 0.08–0.18 Å s^−1^, respectively. Finally, Al electrodes were deposited using a thermal evaporation system through a shadow mask to form an active device area of 1 mm^2^ under a high vacuum of 1 × 10^−4^ Pa. The top mirror of low-Q and intermediate-Q microcavity device consists of 100 nm-thick Al, which was deposited in a vacuum of 2 × 10^−4^ Pa by thermal evaporation with deposition rates of 3.0-5 Å s^−1^. The top mirror of high-Q microcavity device consists of 6.5 pairs of periodic ZnS (63.9 nm-thick)/MgF_2_ (99.5 nm-thick). The ZnS and MgF_2_ layers were deposited in a vacuum of 1 × 10^−3^ Pa by electron beam evaporation with deposition rates of 3.5–4.0, 3.0–3.5, and 2.0–2.5 Å s^−1^, respectively. The electron beam currents of evaporating ZnS and MgF_2_ were 10–13 mA and 25–32 mA.

### Measurement and characterization

The amplitude for magneto-EL and magneto-PL is defined by the relative change in percentage: ${\mathrm{{MFE}}} = \frac{{I_{\mathrm{B}} - I_{\mathrm{0}}}}{{I_{\mathrm{0}}}}$, where *I*_B_ and *I*_0_ are the signal intensities with and without an external magnetic field. The magneto-EL and magneto-PL measurements were performed by using SPEX Fluorolog 3 spectrometer combined with an electrically controllable magnet. Specifically, the magneto-PL and magneto-PL were measured by recording the emission intensity at the spectral peak wavelength as a function of magnetic field. The electrical excitation source was provided by Keithley-2400. The photoexcitation was provided by a 405 nm continuous wave (CW) laser. The EL and PL spectra were measured by SPEX Fluorolog 3 spectrometer. The linearly polarized emission light was detected by putting a linear polarizer after the devices and recording the emission light intensity change with rotating linear polarizer. The EQE were measured by using an absolute EQE measurement system with an integrating sphere. PL lifetimes were measured by using FLS920 time-corrected single photon counting system at an excitation wavelength of 450 nm and an emission wavelength of 556 nm. All the experimental measurements were carried out with encapsulated devices at room temperature.

## Supplementary information

Supplementary Information

## Data Availability

All data supporting the finding of this study are available from the authors upon reasonable request.
