# Supplementary material for: Investigating underlying mechanism in spectral narrowing phenomenon induced by microcavity in organic light emitting diodes
Source: Nat Commun. 2019 Apr 8;10:1614. doi: 10.1038/s41467-019-09585-0 (PMC6453918; doi:10.1038/s41467-019-09585-0)
Supplement: Supplementary file 1 — Supplementary Information [file 41467_2019_9585_MOESM1_ESM.pdf]

Supplementary Information

Investigating Underlying Mechanism in Spectral Narrowing Phenomenon Induced by Microcavity in Organic Light Emitting Diodes

Wang *et al*

Supplementary Figures

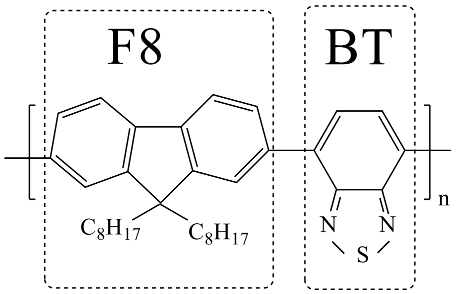

Supplementary Figure 1. Chemical structure of F8BT polymer with F8 and BT moieties.

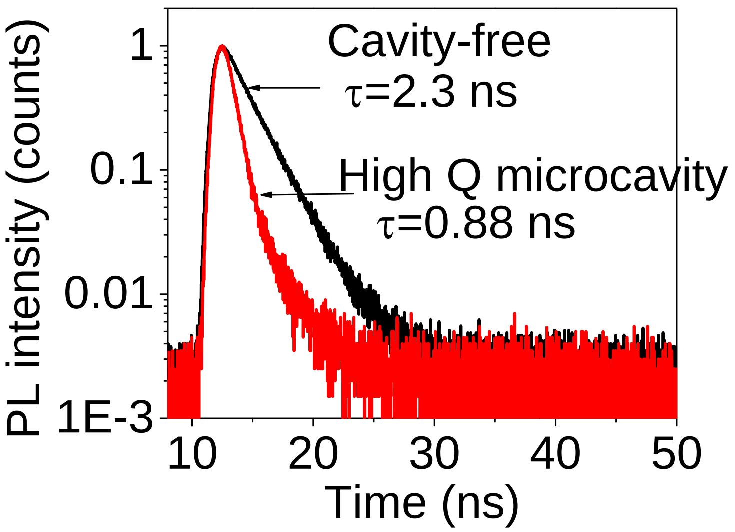

Supplementary Figure 2. The photoluminescence (PL) decay curves of F8BT film measured with and without microcavity.

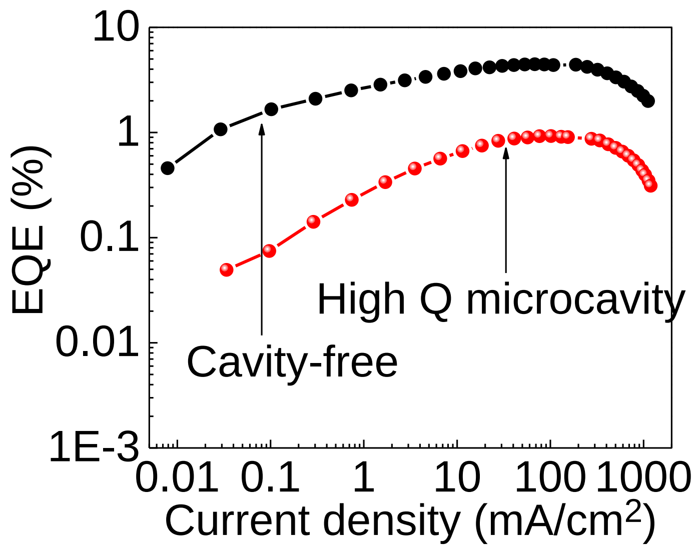

Supplementary Figure 3. External quantum efficiencies (EQE). The EQE was measured for high quality factor (high-Q) cavity-based and cavity-free F8BT organic-light emitting diodes (OLEDs) at different injection currents.

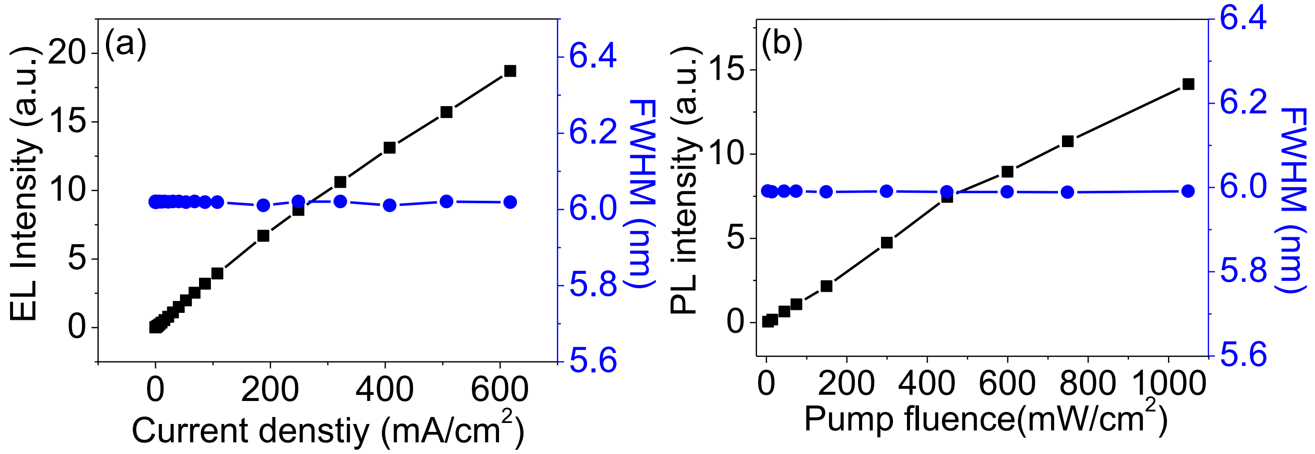

Supplementary Figure 4. Light-emitting intensity and full width at half maximum (FWHM) as function of pumping intensity in high-Q cavity-based F8BT OLED. (**a**) electroluminescence (EL). (**b**) photoluminescence (PL) (Excitation: continuous wave 405 nm laser).

Supplementary Note 1

Discussion on mechanism of magneto-PL and magneto-EL: Specifically, a magnetic field can introduce a coherent spin precession with the frequency (named as Larmor frequency) on electron and hole within a polaron pair. This coherent spin precession can enhance the spin-conserving manor between singlets and triplets and consequently suppresses the spin mixing from singlets to triplets, leading to a positive magneto-PL and magneto-EL when spatially extended states such as polaron pairs or electron-hole pairs or charge-transfer states are formed. This has been shown in organic materials [1,2]. We should point out that, in order to realize magneto-PL and magneto-EL, two time constants: spin lifetime and PL lifetime, must be matched. Once the coherent spin precession introduced by a magnetic field in a polaron pair can be relaxed by internal magnetic interaction from hyperfine interaction or spin-orbital coupling, leading to a spin lifetime. If the spin lifetime is much shorter than the PL lifetime, the introduced spin precession becomes completely relaxed before the PL occurs, disabling magneto-PL and magneto-EL. Only if the spin lifetime is comparable with PL lifetime, the introduced coherent spin precession can still exist before the PL occurs, enabling magneto-PL and magneto-EL.

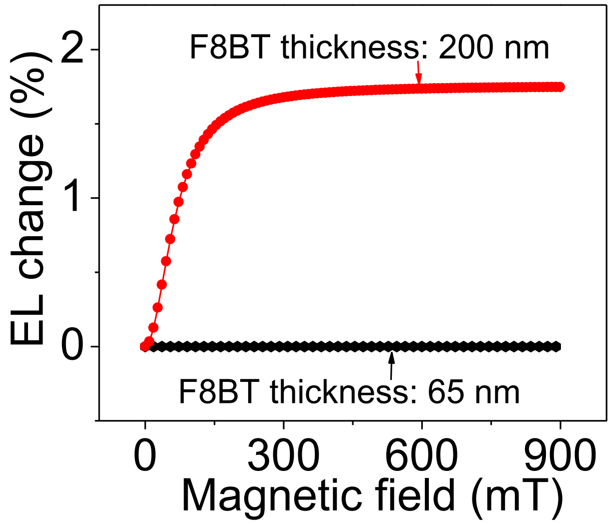

Supplementary Figure 5. Magneto-electroluminescence (magneto-EL). Appreciable and negligible magneto-EL signals from thicker (200 nm) and thinner (65 nm) cavity-free F8BT OLEDs at constant current (20 mA cm^{-2}).

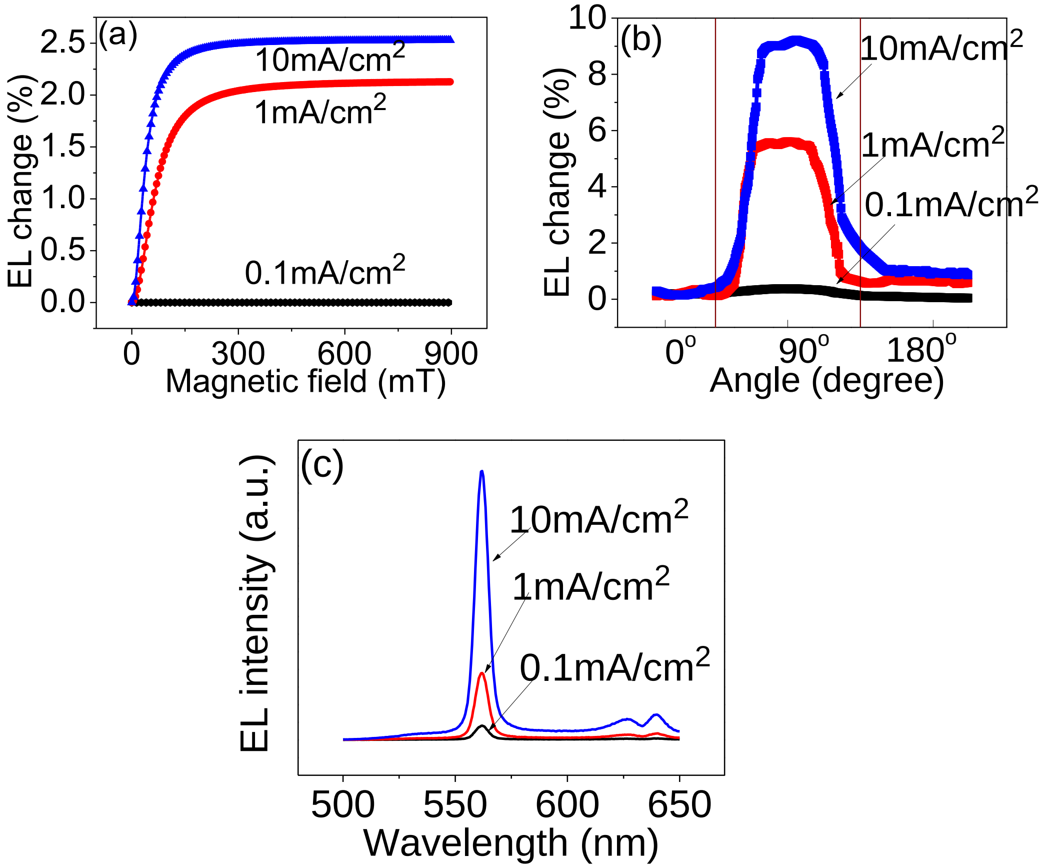

Supplementary Figure 6. Electroluminescence (EL) characterization. (a) magneto-EL, (b) polarized EL output, and (c) EL spectra at different injection current densities for high-Q cavity-based F8BT OLED.

Supplementary Note 2

We can see that the magneto-EL signal and the polarization of EL output are concurrently occurred. Before the EL becomes a linearly polarized output at the injection current lower than 0.1 mA/cm^2, no magneto-EL can be observed even the EL shows a strong intensity at this injection. Only if the EL becomes a linearly polarized output at the injection current higher than 0.1 mA/cm^2, magneto-

EL can be clearly observed in high-Q microcavity device. This study confirms that the new excited states with spatially extended wavefunctions with characteristics similar to polaron pairs are formed and become aligned within the microcavity to generate a narrow EL. This provides a promising precondition to develop electrically pumped lasing devices based on this microcavity design.

Supplementary References

1. Bergeson, J., Prigodin, V., Lincoln, D. & Epstein, A. Inversion of magnetoresistance in organic semiconductors. *Phys. Rev. Lett.* **100**, 067201 (2008).

2. Bobbert, P., Nguyen, T., Van Oost, F., Koopmans, v. B. & Wohlgenannt, M. Bipolaron mechanism for organic magnetoresistance. *Phys. Rev. Lett.* **99**, 216801 (2007).
